# Recent Advances in Modeling Mitochondrial Cardiomyopathy Using Human Induced Pluripotent Stem Cells

**DOI:** 10.3389/fcell.2021.800529

**Published:** 2022-01-10

**Authors:** Mario G. Pavez-Giani, Lukas Cyganek

**Affiliations:** ^1^ Stem Cell Unit, Clinic for Cardiology and Pneumology, University Medical Center Göttingen, Göttingen, Germany; ^2^ German Center for Cardiovascular Research (DZHK), Partner Site Göttingen, Göttingen, Germany; ^3^ Cluster of Excellence “Multiscale Bioimaging: From Molecular Machines to Networks of Excitable Cells”, University of Göttingen, Göttingen, Germany

**Keywords:** mitochondrial cardiomyopathy, mitochondrial disease, mtDNA, heteroplasmy, induced pluripotent stem cells (hiPSCs), iPSC-derived cardiomyocytes

## Abstract

Around one third of patients with mitochondrial disorders develop a kind of cardiomyopathy. In these cases, severity is quite variable ranging from asymptomatic status to severe manifestations including heart failure, arrhythmias, and sudden cardiac death. ATP is primarily generated in the mitochondrial respiratory chain via oxidative phosphorylation by utilizing fatty acids and carbohydrates. Genes in both the nuclear and the mitochondrial DNA encode components of this metabolic route and, although mutations in these genes are extremely rare, the risk to develop cardiac symptoms is significantly higher in this patient cohort. Additionally, infants with cardiovascular compromise in mitochondrial deficiency display a worse late survival compared to patients without cardiac symptoms. At this point, the mechanisms behind cardiac disease progression related to mitochondrial gene mutations are poorly understood and current therapies are unable to substantially restore the cardiac performance and to reduce the disease burden. Therefore, new strategies are needed to uncover the pathophysiological mechanisms and to identify new therapeutic options for mitochondrial cardiomyopathies. Here, human induced pluripotent stem cell (iPSC) technology has emerged to provide a suitable patient-specific model system by recapitulating major characteristics of the disease *in vitro*, as well as to offer a powerful platform for pre-clinical drug development and for the testing of novel therapeutic options. In the present review, we summarize recent advances in iPSC-based disease modeling of mitochondrial cardiomyopathies and explore the patho-mechanistic insights as well as new therapeutic approaches that were uncovered with this experimental platform. Further, we discuss the challenges and limitations of this technology and provide an overview of the latest techniques to promote metabolic and functional maturation of iPSC-derived cardiomyocytes that might be necessary for modeling of mitochondrial disorders.

## Introduction

Mitochondrial diseases comprise a multisystemic group of metabolic disorders characterized by early- or late-onset progressive neurodegenerative and cardiac symptoms, likely associated with a psychomotor regression, encephalopathy, myopathy and cardiomyopathy (DiMauro and Schon, 2013). Around 20–40% of children with a mitochondrial disease develop a kind of cardiac symptoms (Brambilla et al., 2020; Mazzaccara et al., 2021). In such cases, severity is quite variable ranging from asymptomatic status to severe manifestations including heart failure, arrhythmias, and sudden cardiac death. The highest prevalence in these patients is to develop a hypertrophic cardiomyopathy (HCM), nonetheless, there are also reports about cases with mitochondrial-caused restrictive, dilated and left ventricular non-compaction cardiomyopathies (Finsterer and Kothari, 2014). As a result of its continuous contractile work, the heart is an extremely oxidative organ which demands a high amount of energy. To sustain this task, the adult human heart consumes 6 kg of adenosine triphosphate (ATP) each day (Taegtmeyer, 1994; Bottomley et al., 1996). ATP is primarily generated in the mitochondrial respiratory chain (MRC) by the oxidative phosphorylation system (OXPHOS) by utilizing fatty acids and carbohydrates (Kolwicz et al., 2013). If the energy production in the heart is inhibited, the cardiac capacity to store ATP is only sufficient to sustain three heart beats, making the heart extremely vulnerable to mitochondrial insufficiency (Ingwall, 2009). Indeed, a deleterious mitochondrial function can detrimentally impact the contraction or relaxation capacity of the heart (Brown et al., 2017).

Mitochondria is a ubiquitous double membrane organelle that contains its own genome, which is maternally inherit and presents in multiple copies in every eukaryotic cell. Around 95% of the ATP production in cardiomyocytes is produced by substrate oxidation in the mitochondria (Doenst et al., 2013; Rosca and Hoppel, 2010). The OXPHOS is localized in the inner mitochondrial membrane where several metabolic pathways converge, with β-oxidation and Krebs cycle (citric acid cycle) being the most relevant in cardiomyocytes (Lai et al., 2014). Subsequently, both routes deliver nucleotides to Complex I (NADH-ubiquinone oxidoreductase) and Complex II (succinate dehydrogenase) in the MRC; nicotinamide-adenine-dinucleotide (NADH) delivered by Krebs cycle, and flavin-adenin-dinucleotide (FADH_2_) from β-oxidation, respectively (Brandt, 2006; Cecchini, 2003; Gu et al., 2016). Complex III (ubiquinol-cytochrome c oxidoreductase) represents the central core for the MRC, reducing cytochrome c from coenzyme Q oxidation. Here, the so-called Q-cycle mechanism takes place, which ensures a continuing proton pumping from the matrix to the intermembrane space (Slater, 1983). Finally, each cytochrome c molecule delivers one electron into the Complex IV (cytochrome c oxidase), which transfers them to one dioxygen molecule, converting the molecular oxygen into two molecules of water (Turrens, 2003). Consequently, three proton translocating complexes (Complex I, Complex III and Complex IV), connected by the mobile electron carrier ubiquinone and cytochrome c, catalyze the electron transfer from NADH to O_2_, thereby promoting an electrochemical gradient among the inner space membrane and the mitochondrial matrix. This gradient is used by Complex V (or adenosine triphosphate synthase; ATP synthase), to generate ATP from ADP conversion (Neupane et al., 2019). Notably, the MRC can exist as distinct enzymes or associate in supercomplexes that may facilitate its stabilization as well as the provision of greater spatiotemporal control of respiration (Vercellino and Sazanov, 2021).

**FIGURE 1:**
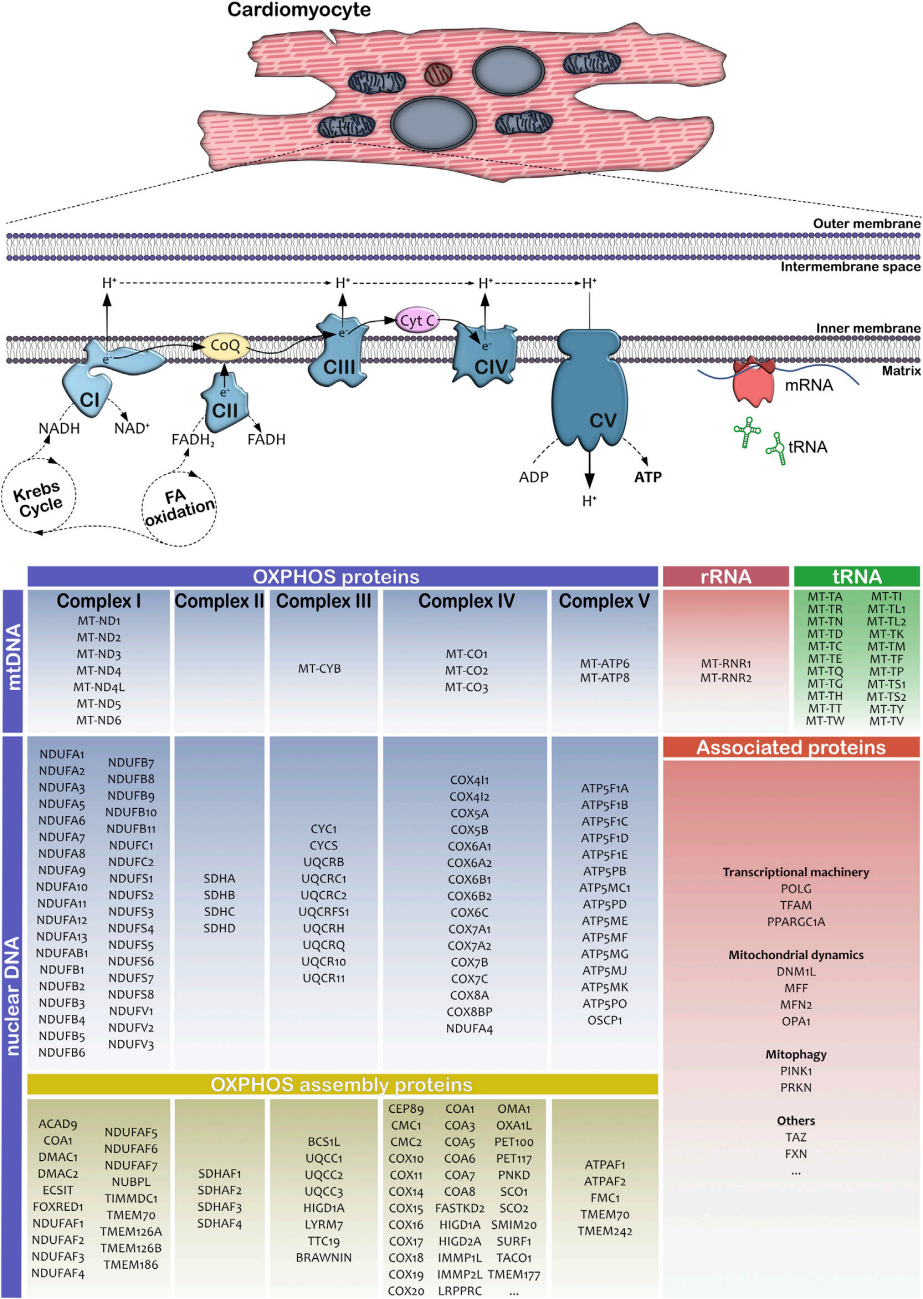
Schematic representation of mitochondria-associated genes involved in protein synthesis and complex formation of the oxidative phosphorylation and ATP synthesis. The table summarizes the most important OXPHOS subunit and assembly proteins in human cells. mtDNA indicates mitochondrial DNA; nDNA, nuclear DNA; rRNA, ribosomal RNA; tRNA, transfer RNA; FA, fatty acids; CI, Complex I or NADH ubiquinone oxidoreductase; CII, Complex II or succinate dehydrogenase; CIII, Complex III or ubiquinol-cytochrome c oxidoreductase; CIV, Complex IV or cytochrome c oxidase; CV, Complex V or ATP synthase; CoQ, coenzyme Q10 and Cyt C, cytochrome c.

OXPHOS is encoded by genes in both the mitochondrial (mtDNA) and the nuclear genome, and in fact, genes on both genomes are critical in controlling the bioenergetic mitochondrial function (Smeitink et al., 2001). Maternally inherited mtDNA is circular and relatively small (over 16,000 base pairs) and encodes 37 genes: 13 proteins, 22 transfer-RNAs and two ribosomal RNAs (Figure 1). Although this group of genes represents only 1% of mitochondrial proteins, it is critical for a proper OXPHOS function (Anderson et al., 1981; Schon et al., 2012). mtDNA is localized in the mitochondrial matrix, closely to the MRC, one of the major sources for reactive oxygen species (ROS) (Bogenhagen, 2012). Hence, the mitochondrial genome is highly susceptible for spontaneous mutations. In fact, epidemiological studies demonstrated that mutations in mitochondria-related genes including single-nucleotide variants to large-scale deletions, affect 1 in 4,500 individuals (Chinnery et al., 2000), and the risk to develop cardiac symptoms is significantly higher in this patient cohort. Furthermore, a clinical study showed that 36% of patients with mitochondrial disorders harboring mutations in nuclear DNA- or mtDNA-encoded genes developed a kind of cardiomyopathy (Brambilla et al., 2020), probably due to the high bioenergetic demand required by the heart. At the same time, infants with cardiovascular compromise in mitochondrial deficiency display a worse late survival compared to patients without cardiac compromise and current therapies are unable to substantially mitigate the disease burden (Wahbi et al., 2015; Brambilla et al., 2020). The genotype-phenotype correlation in patients with mitochondrial cardiomyopathies as well as the underlying pathophysiological mechanisms associated to mitochondrial gene mutations are still poorly understood. This is particularly true when it comes to understanding the impact of mtDNA gene mutations, mainly because the variable amount of pathogenic mtDNA variant load (heteroplasmy) represents an additional level of complexity in the interpretation of clinical and genetic findings.

In the present review, we will explore the advantages and challenges of human induced pluripotent stem cell (iPSC) technology applied for mitochondrial cardiomyopathies, describing novel findings in modeling mtDNA and nuclear DNA mitochondrial gene mutations, as well as its potential scopes for biomedical research and therapeutic approaches.

## Human Induced Pluripotent Stem Cells as Cardiomyopathy Model

Cardiomyopathy is a clinically heterogeneous group of cardiac disorders that can be classified in congenital (or inherited), acquired or mixed subtypes, with the latter one accounting for individuals that carry distinct genetic risk factors that might provoke cardiac symptoms under certain environmental circumstances (Elliott et al., 2014). Historically, cardiomyopathies have been classified in hypertrophic cardiomyopathy (HCM), dilated cardiomyopathy (DCM), and arrhythmogenic right ventricular cardiomyopathy (ARVC), nonetheless, due the upgrade of genetic and molecular assays, a better understanding has been gained in terms of etiology and phenotypic expression (Brieler et al., 2017). Congenital cardiomyopathy is caused by genetic mutations that affect the heart and/or the cardiovascular system. Whereas a certain correlation exists between the clinical features of cardiomyopathies and an autosomal dominant inheritance of gene mutations—as it is for instance known for MYH7 gene mutations causing HCM or titin truncating variants leading to DCM (Herman et al., 2012; Marian and Braunwald, 2017)—the etiology of around 40% of the cases with cardiac symptoms remains unknown (Campuzano et al., 2014).

Over the last years, the vast development of iPSC technology allows to study human diseases by using patient-specific cells *in vitro*. By activation of four essential transcriptional factors (such as OCT4, SOX2, C-MYC, and KLF4), isolated somatic cells from skin biopsies or blood samples from donors can be reprogrammed into iPSCs that harbor the identical genome of the donor including genetic variants/defects (Robinton and Daley, 2012). Significant progress has been made in developing efficient protocols for the directed differentiation of iPSCs into functional cardiomyocytes (iPSC-CMs), thus we are now able to generate defined cardiac subtypes in high quantity and purity. Substantial evidence shows that iPSC-CMs derived from patients with congenital cardiomyopathies represent the phenotype of the disease, thereby providing a unique and powerful platform for modeling genetic diseases as well as investigating the underlying pathological mechanisms.

Nowadays, iPSC-derived cardiomyocytes have been applied to gain insights into channelopathies (such as Long-QT and Brugada syndrome) (Garg et al., 2018), sarcomeric cardiomyopathies (such as HCM and DCM) (Eschenhagen et al., 2015), syndromic cardiomyopathies (such as Noonan syndrome or Duchenne muscular dystrophy) (Long et al., 2018; Hanses et al., 2020), or polygenetic cardiac diseases (Borchert et al., 2017). However, although many phenotypic properties can be recapitulated in patients’ iPSC-CMs, a higher ‘adult-like’ maturation state and appropriate physiological conditions might to be required to comprehensively mimic all aspects of the disease in the dish. Therefore, pursuing efforts are being made to accomplish suitable mature conditions, which involve, among others, energetic substrates delivered in culture medium (Yang et al., 2019), growth factors (Yang et al., 2014), metabolic pathway modulators (Gentillon et al., 2019; Sarikhani et al., 2020) and engineered 3D cardiac tissue (Tiburcy et al., 2017). Further, this technology is now gradually entering into preclinical and clinical phase by testing of novel therapeutic options, such as screening of chemical compounds or evaluating gene therapy approaches (Musunuru et al., 2018).

In the following sections, we summarized the promises and challenges of iPSC-based modeling of mitochondrial cardiomyopathies, involving both nuclear and mitochondrial genome mutations, as well as the analysis of bioenergetic disturbances as a consequence of heart failure via iPSC-CMs.

## Modeling Mitochondrial Cardiomyopathies Using iPSC-Derived Cardiomyocytes

Mitochondrial disorders triggered by both nuclear DNA- and mtDNA-encoded mutated genes might cause a kind of cardiomyopathy. For a long time, the absence of robust and reliable mitochondrial disease models hindered to explore the underlying pathomechanisms and to find new treatment options for this rare condition, principally due to the genetic heterogeneity of patients and challenges to recapitulate the disease phenotypes in animal models due to the interspecies discrepancy in cardiac physiology (Chanana et al., 2016; Ulmer and Eschenhagen, 2020). Here, modeling of mitochondrial DNA-encoded gene mutations represents a particular difficulty, as it is virtually impossible to introduce a desired gene variant in a multitude of copies of the mitochondrial genome. Most of the strategies to generate a disease models with mtDNA variants involved transgenic animals in combination with cytoplasmic transplantation (so-called cybrids) (Wilkins et al., 2014). Although these models might display some of the metabolic features, they are not able to fully recapitulate the patients’ genetic context and therefore cannot represent the entire picture of the disease.

Recent reports exploited iPSC-CMs as platform to overcome this challenge by reprogramming patients’ somatic cells. Throughout this approach, novel investigations were made possible in analyzing the key molecular and functional features of patients’ cardiomyocytes and revealing novel insights in cardiac disease progression of mitochondrial disorders. Herein, we will highlight some of the major findings obtained using iPSC-CMs and we will explore the pathophysiological causality of both mtDNA and nuclear DNA gene mutations in causing mitochondrial cardiomyopathy.

### mtDNA Disorders

**FIGURE 2:**
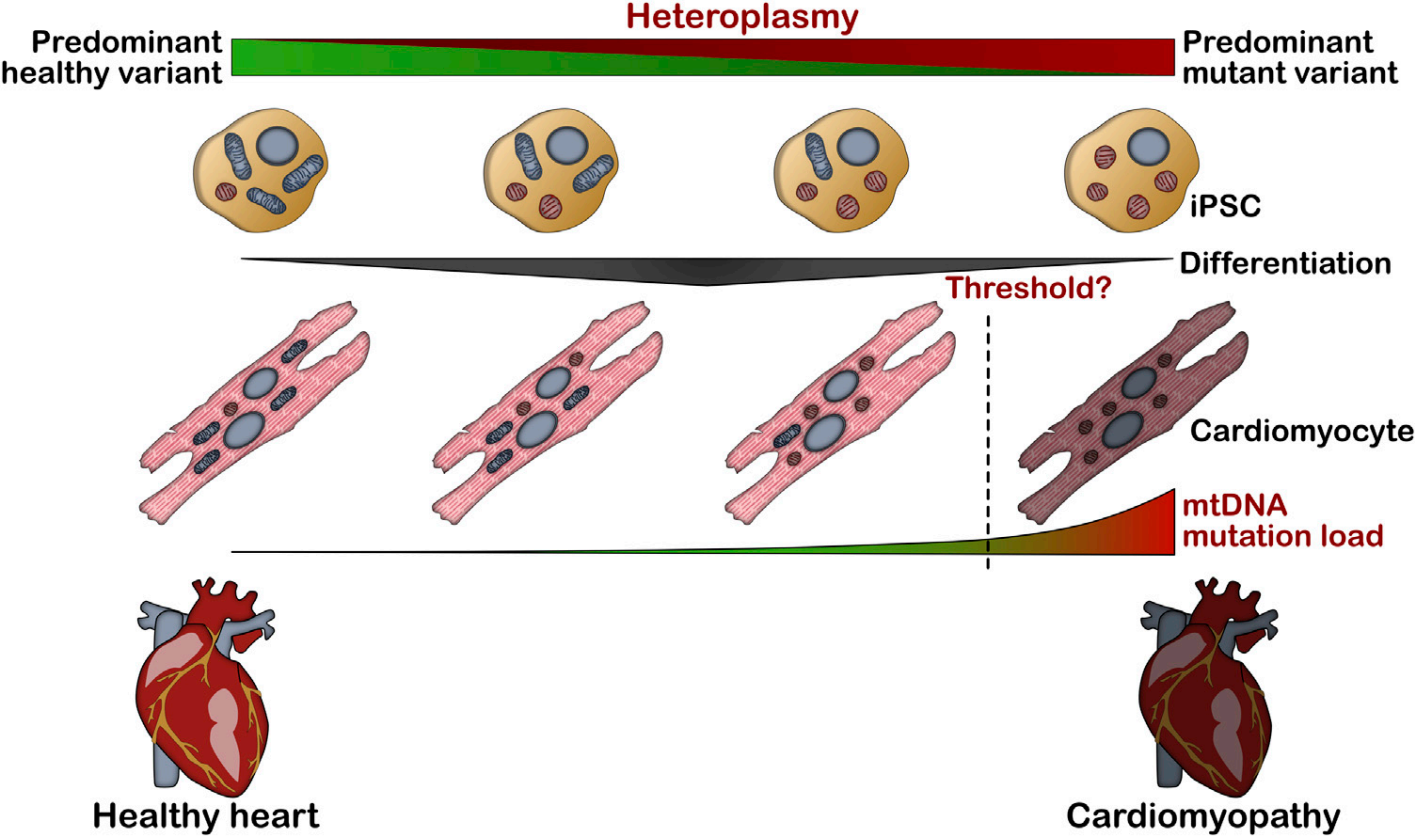
Impact of mtDNA heteroplasmy levels on cardiomyocyte pathology. mtDNA heteroplasmy associated to pathogenic variant load in iPSCs is typically preserved during cardiomyocyte differentiation, promoting poor mitochondrial performance and cardiac remodeling when mtDNA variant load exceeds a certain.

The involvement of mitochondrial dysfunction as a result of mutations in mtDNA-encoded genes was first observed more than 3 decades ago. Wallace et al. (1988) provided the first approximation through identifying a point mutation in the mtDNA gene for subunit 4 of the NADH dehydrogenase complex (MT-ND4) to be associated with maternally inherited Leber’s hereditary optic neuropathy. Since then, a completely new genomic mitochondrial era was pioneered, describing many other syndromes related to this gene. In eukaryotic cells, nuclear DNA and mtDNA co-exist, based on the cell type, in a ratio of 1:10 in sperm, over 1:100–300 in stem cells/iPSCs up to 1:1,000–10,000 in terminally differentiated cells such as cardiomyocytes, in line with an increased organelle biogenesis upon differentiation (Kelly et al., 2012; Kelly et al., 2013) Due to spontaneous variations in the mitochondrial genome, mtDNA within one cell might be mixture of different mtDNA genomes, and this event is called heteroplasmy (Stewart and Chinnery, 2015). When a mixture of normal and mutated mtDNA is present, the cell may randomly deliver these variations to daughter cells (Stewart and Chinnery, 2021). As a consequence of this event, daughter cells can receive a different load of mutant mtDNA, generating diverse levels of heteroplasmic mutations (Durham et al., 2007). Considering that 1) this event occurs during every cell division, and 2) mtDNA copy number from less than 300 copies in undifferentiated cells significantly increases as cells differentiate into mature functional cells the percentage of mutant mtDNA load might highly differ between tissues and organs (Figure 2). However, when mtDNA variant load exceeds a certain threshold (which might highly differ between individual gene mutations), some clinical manifestations in certain tissues may occur (Yu-Wai-Man et al., 2010). This phenomenon, in addition to the unique patient’s genetic background, explains the high range of signs and symptoms that are depicted in mtDNA disorder patients.

An additional challenge represents the variable heteroplasmy levels in biopsy material used for iPSC generation (Perales-Clemente et al., 2016). However, the variable amount of mutant mtDNA load in somatic cells (such as dermal fibroblasts) allows to generate patient-specific iPSC lines with high and low heteroplasmic levels in parallel during one reprogramming procedure (Kodaira et al., 2015; Ma et al., 2015). Further, once a iPSC line is established, mtDNA heteroplasmy levels are not significantly altered during *in vitro* differentiation, so that higher and lower percentage of heteroplasmy is preserved in fully differentiated cells (Folmes et al., 2013; Klein Gunnewiek et al., 2020). In-depth genetic screening methods nowadays allow a detailed estimation of the heteroplasmy levels in patients, further enhancing our knowledge of the impact of mtDNA variants in mitochondrial disorders. However, there is still little knowledge about the consequences of mtDNA mutations in the manifestation of disease symptoms and, so far, only few studies investigated the cardiac phenotype of mtDNA mutations via patient-specific iPSC-CMs.

For instance, Leigh syndrome is a multi-systemic disease characterized by a progressive neurodegenerative disorder, as a result of OXPHOS gene mutations encoded either by the mitochondrial or nuclear genome (Sofou et al., 2018). The mitochondrial gene mutation m.13513G>A within the MT-ND5 gene, encoding the core subunit of Complex I, has been reported as a frequent cause of Leigh syndrome and is commonly associated with cardiac defects (Ruiter et al., 2007). Galera-Monge et al. (2019) generated iPSCs from patients’ fibroblasts with high (>40%) and low (<20%) mutation load of the respective m.13513G>A variant. Surprisingly, high mutation load iPSCs displayed a highly impaired cardiac differentiation, probably due to an ineffective epithelial-mesenchymal transition at the first days of differentiation. Nevertheless, when using iPSCs with a mtDNA mutation load below the threshold of 30%, dysfunctional iPSC-CMs could be obtained which presented aberrant pattern of beating strength and rate and higher apoptosis levels compared to the control group in a mutant-load dependent way (Galera-Monge et al., 2019).

A similar outcome was obtained from in an iPSC model from a patient with mitochondrial encephalomyopathy and stroke-like episodes (MELAS) (Yokota et al., 2017). Patient-specific iPSCs with high (>90%), intermediate (80–90%) and low (40–50%) mutation load were employed to explore the cardiac and neurological consequences caused by the heteroplasmic mitochondrial gene mutation m.3243A>G within MT-TL1 (encoding a mitochondrial transfer RNA). By analyzing skin fibroblasts collected from MELAS patients, the molecular pathogenic threshold for this particular mutation with regard to mitochondrial respiratory function was expected to be around 90% (Yokota et al., 2015). In contrast to intermediate and low mutation load, and in line with the neuronal differentiation of these lines, the mitochondrial dysfunction promoted by high-load m.3243A>G disturbed cardiac lineage commitment during iPSC differentiation, suggesting that the mitochondrial protein synthesis machinery is central for triggering cardiomyocyte differentiation (Yokota et al., 2017). In line, iPSC-CMs from two patients with myoclonic epilepsy with ragged red fiber (MERRF) syndrome harboring a mutation in a different mitochondrial transfer RNA, namely the mitochondrial gene mutation m.8344A>G in MT-TK, and here with a mutation load around 50%, revealed an impaired mitochondrial respiration, an abnormal ultrastructure of mitochondria, elevated levels of ROS, as well as upregulation of antioxidant genes (Chou et al., 2016).

A different study demonstrated that iPSC-CMs derived from patients with a family history of maternally inherited HCM, who harbor the homoplasmic mitochondrial gene mutation m.2336T>C in MT-RNR2 (100% mutated mtDNA load), encoding the large subunit of the mitochondrial ribosomal RNA, resulted in mitochondrial dysfunction and ultrastructural defects by decreasing the stability of the 16S ribosomal RNA, which consequently led to reduced levels of mitochondrial proteins. Furthermore, mtDNA variants can lead to energetic depletion through impaired OXPHOS and mitochondrial Ca^2+^ regulation, which are central for excitation–contraction coupling in cardiac cells (Sommakia et al., 2017). Indeed, iPSC-CMs that harbor the m.2336T>C variant also presented disturbances in cytosolic Ca^2+^ handling and a deleterious electrophysiological pattern, thereby possibly triggering the manifestation of HCM in these patients (Li et al., 2018). Interestingly, by combining mtDNA sequencing data from a cellular iPSC-CM model of HCM with genetic screens from multiple unrelated HCM patients, novel potentially HCM-protective and HCM-deteriorating mtDNA variants were identified, mostly not overlapping with common ones in mitochondrial diseases (Kargaran et al., 2020).

The number of iPSC-based reports within the last decade underlines the challenges that are associated with the modeling of mtDNA gene mutations and the impact of heteroplasmy. Given the advantage of iPSC-CMs over other model systems, e.g. by allowing to precisely investigate the genotype-phenotype correlation in cardiac cells with different heteroplasmic load, iPSC-CM disease modeling might be considered a highly suitable model to uncover novel insights in the underlying pathomechanisms related to mtDNA gene variants.

### Nuclear DNA Disorders

Nuclear DNA-encoded proteins represent around 99% of the total mitochondrial proteome and due the diversity of function that mitochondria exhibit, variations in this gene group extend beyond bioenergetic perturbations. Proteomic studies have revealed that mitochondria harbor almost 1,500 different proteins (Prokisch et al., 2004; Morgenstern et al., 2017; Pfanner et al., 2019), that are related, among others, to biosynthesis of amino acids, lipids, heme and Fe–S clusters, cellular signaling pathways, regulation of Ca^2+^ levels, quality control and programmed cell death.

The generation of iPSC-based models for mitochondrial disorders such as Leigh syndrome is not dissimilar to any other iPSC model when it comes to nuclear DNA mutations. However, the majority of studies have focused on the neurological phenotyping of these patient lines, probably since neuro-related symptoms are a major hallmark of mitochondrial disorders (Zhao et al., 2018; Liang et al., 2020; Inak et al., 2021). Most of the iPSC-CM studies related to nuclear-encoded gene mutations in OXPHOS have been focused in Complex IV deficiency, which has been involved in certain forms of inherited cardiomyopathies (Jaksch et al., 2000). Briefly, the main function of Complex IV is to oxidize cytochrome c and to transfer electrons to molecular oxygen. Here, the mitochondria-encoded COX1 and COX2 provide the essential sites for oxygen reduction, by housing the redox-active heme and copper centers (Capaldi, 1990; Khalimonchuk et al., 2010). Further, nuclear-encoded SCO1, SCO2, and COA6 are required for copper center formation (Leary et al., 2014; Soma et al., 2019). A loss of function of these chaperones and the concomitant cytochrome c oxidase deficiency causes severe human disorders (Brischigliaro and Zeviani, 2021). SCO2 encodes a 266 amino acid metallochaperone that participate in copper delivery to COX, and its mutations represent the most common cause of COX or Complex IV deficiency, often leading to cardiomyopathies (Jaksch et al., 2000). The first study using iPSC-CMs to model COX deficiency was conducted by Hallas et al. (2018) by generating iPSCs from two Leigh syndrome patients with HCM harboring the homozygous variant SCO2^G193S^ (c.577G>A) and the compound-heterozygous variant SCO2^E140K^ (c.418G>A) in combination with an early truncating variant (c.17ins19), respectively. In the study, the SCO2-associated COX deficiency resulted in ultrastructural abnormalities of the mitochondria, a reduced mitochondrial oxidative ATP production capacity, as well as aberrant Ca^2+^ handling and an arrhythmic contractility pattern in the patients’ iPSC-CMs.

Meanwhile, new reports have proven evidence of mitochondrial disease models harboring gene mutations in accessorial components of OXPHOS. Friedreich’s ataxia is autosomal recessive genetic condition that causes a neuro-degenerative movement disorder, with a typical age of onset between 10 and 15 years (Koeppen, 2011), and HCM is quite prevalent in this patient cohort (Weidemann et al., 2013). The condition is caused by expanded GAA repeats within the first intron of the nuclear-allocated gene frataxin, a protein that is involved in the biosynthesis of iron-sulfur co-factors required by multiple mitochondrial and extra-mitochondrial proteins (Castro et al., 2019). One of the first publications investigating the cardiac consequences of the Friedreich’s ataxia syndrome via patients’ iPSCs was described in 2013 (Hick et al., 2013). In the aforementioned work, the group applied iPSCs from two patients and registered partially degenerated mitochondria with abnormal structures—in line with mitochondrial damages observed in cardiac tissues from Friedreich’s ataxia patients—suggesting a functional defect in cellular respiration in these cells. Lee et al. (2016) revealed increased ROS levels, intracellular iron accumulation, as well as electrophysiological and Ca^2+^ handling abnormalities in patients’ iPSC-CMs . Another report provided evidence for the presence of an HCM-dependent transcriptional profile in cells from Friedreich’s ataxia patients (Li et al., 2019). Furthermore, a recent study proved that the contractile capacity from both patient-derived and frataxin-knockdown iPSC-CMs showed a poor performance using human engineered tissue modeling (Wong et al., 2019). In depth, frataxin-deficient cardiac tissues displayed attenuated force development compared to healthy controls with a correlation between frataxin gene expression and contractility. In addition, an aberrant electrophysiological pattern was detected (depicted as prolongation of the action potential duration and reduction of the maximum capture rate), thereby recapitulating some major clinical findings (Wong et al., 2019).

Similar efforts had been conducted in modeling Barth syndrome, a rare X-linked genetic disorder characterized by cardiomyopathy, skeletal myopathy, growth delay, neutropenia and increased urinary excretion of 3-methylglutaconic acid (Clarke et al., 2013). This disease has been mainly described by mutations in the nuclear-encoded tafazzin (Bione et al., 1996). Barth syndrome presents a unique pathophysiological mechanism: in detail, mutated tafazzin primary promotes an aberrant cardiolipin maturation process due its role in the transacylation process showing preference for the transfer of a linoleic acid group from phosphatidylcholine to monolysocardiolipin, resulting in depletion of mature cardiolipin and accumulation of an immature form and thereby in a deleterious mitochondrial metabolism (Garlid et al., 2020). Cardiolipin is an important component of the inner mitochondrial membrane, where it constitutes about 20% of the total lipid composition and affects many aspects of mitochondrial structure and function, including MRC complex formation/interaction, mitochondrial dynamics, and apoptosis (Dudek, 2017). In an extensive study, Wang et al. (2014) generated patient-specific iPSC lines with a tafazzin frameshift mutation (c.517delG) and a missense mutation TAZ^S110P^ (c.328T>C), respectively, as well as different CRISPR/Cas9-engineered tafazzin-deficient iPSC lines, in order to investigate the mitochondrial cardiomyopathy *in vitro*. Barth syndrome cells recapitulated the expected muscle-selective phenotype with an impaired cardiolipin biogenesis (by exceeding the monolysocardiolipin-to-cardiolipin ratio), smaller and fragmented mitochondria, deficits in the oxygen consumption rate, a decreased efficiency in ATP generation, as well as higher levels of ROS. Further, by analyzing iPSC-CM-based engineered cardiac tissues, tafazzin deficiency revealed impaired sarcomere assembly and defects in contractility that occurred independently of ATP depletion, thereby suggesting that both proper mitochondrial function and reduced ROS production is crucial for sarcomerogenesis and contractile performance (Wang et al., 2014). In a subsequent study, the group identified aberrant Ca^2+^ handling as major cause for the contractile deficits in the patients’ cells, most probably due to ROS-dependent activation of Ca^2+^/calmodulin-dependent protein kinase II and subsequent phosphorylation of the ryanodine receptor 2 (Liu et al., 2021). The identical patient line with the frameshift mutation was further used to identify an accumulation of cellular long chain acylcarnitines, as well as alterations in metabolic pathways related to energy production in iPSC-CMs (Fatica et al., 2019). In agreement with the previous reports, Dudek et al. reported a significant downregulation in the mitochondrial bioenergetic capacity in iPSC-CMs from a patient harboring the missense mutation TAZ^G197V^ (c.590G>T) (Dudek et al., 2016). Interestingly, the detrimental mitochondrial respiration coincided with dramatic structural remodeling of MRC supercomplexes and a deficiency in succinate dehydrogenase. Further, cardiolipin remodeling impaired NF-κB signaling and affected the HIF-1α response to hypoxic conditions (Chowdhury et al., 2018).

Moreover, a homozygous intronic variant (IVS3-1G>C) in DNAJC19 (also called TIM14), affecting splicing and resulting in gene loss-of-function, has been described to cause dilated cardiomyopathy with ataxia syndrome (Davey et al., 2006). Due to its similarities in abnormal metabolism, it has been linked to Barth syndrome. TIM14 is predicted to function as a part of the mitochondrial import complex facilitating the import of nuclear-encoded proteins into the mitochondria (Mokranjac et al., 2006), but also to regulate cardiolipin remodeling by tafazzin (Davey et al., 2006). However, by investigating iPSC-CMs from two patients with the respective homozygous variant, no differences in the cardiolipin content were observed (Rohani et al., 2020). Instead, fragmented and abnormally shaped mitochondria were recorded together with an isoform imbalance of OPA1, an important regulator of mitochondrial fusion that has been linked to heart failure.

Lastly, patients with autosomal recessive gene mutations in ACADVL, an inner-mitochondrial membrane localized enzyme that catalyzes the first step of the mitochondrial long-chain fatty acid β-oxidation pathway, possess a very long-chain acyl-CoA dehydrogenase deficiency and possess a high risk for developing cardiac arrhythmias. Consequently, patient-derived iPSC-CMs with a homozygous frameshift variant ACADVL^P35Lfs*26^ (c.104delC) or compound-heterozygous gene mutations ACADVL^V283A^/ACADVL^E381del^ (c.848T>C/c.1141_1143delGAG), respectively, revealed accumulation of potentially toxic intermediates of long-chain fatty acid oxidation (Knottnerus et al., 2020). Further, the cells displayed electrophysiological abnormalities (such as short action potentials), disturbances in Ca^2+^ handling) and presented delayed afterdepolarizations, thereby providing a mechanistic link for the cardiac arrhythmia risk in these patients (Knottnerus et al., 2020; Verkerk et al., 2020).

In summary, nuclear DNA-encoded mitochondrial genes represent 99% of the total mitochondrial proteome and functionality of many of these genes is still unknown. Patient-specific iPSCs could unmask the pathophysiologic mechanisms of several of them. Moreover, since mitochondrial function is involved in many different cellular processes, iPSC-based disease modeling has helped to explore the role of these proteins in other signaling hubs, beyond their importance in energetic homeostasis.

### Mitochondrial Dysfunction as a Consequence of Cardiomyopathies

Metabolic imbalances and bioenergetic perturbations are not only observed in primary mitochondrial disorders but might also be a consequence in other cardiomyopathies related to specific genetic conditions. As an example, phospholamban is a sarco/endoplasmic reticulum Ca^2+^-ATPase pump regulator and an active player in the cytosolic Ca^2+^ handling and contractility (Frank and Kranias, 2000). Carriers with the recurrent mutation PLN^R14del^ (c.40_42delAGA), which is commonly reported in cardiomyopathy patients from Europe and USA (Haghighi et al., 2006; Kayvanpour et al., 2017), present a significantly higher risk to suffer from DCM and arrhythmogenic episodes associated to aberrant Ca^2+^ handling and pathological cardiac remodeling (Posch et al., 2009). A recent study by Cuello and co-workers described a tight correlation between phospholamban gene mutations and mitochondrial dysfunction. Particularly, patients’ iPSC-CMs harboring the PLN^R14del^ variant depicted a detrimental mitochondrial function by displaying a low oxygen consumption rate as well as elevated mitochondrial ROS production (Cuello et al., 2021). Notably, whereas sarcoplasmic reticulum function remained unaltered, impairments of the endoplasmic reticulum to mitochondria crosstalk were detected resulting in lipid accumulation, mitochondrial dysfunction and degeneration, suggesting a cause-consequence correlation provided by a deleterious cytoplasmic Ca^2+^ signaling (Cuello et al., 2021).

In a different study, by generating multiple CRISPR-engineered iPSC lines harboring the HCM-associated gene mutation MYH7^R453C^ (c.9123C>T), Mosqueira et al. (2018) not only recapitulated the classical hallmarks of HCM (such as cellular hypertrophy, sarcomeric disarray or Ca^2+^ handling disturbances), but also identified increased oxygen consumption rates in affected iPSC-CMs. Hence, the authors could show that the mutation-induced sarcomeric disarray caused an inefficient sarcomeric ATP utilization and energy depletion by maintaining the mitochondrial contents unchanged, thereby improving our mechanistic understanding in HCM.

Duchenne muscular dystrophy (DMD) is another genetic condition that causes a severe cardiomyopathy, particularly DCM, due to a loss of dystrophin (Kamdar and Garry, 2016). Patient-derived iPSC-CMs from two patients with out-of-frame deletions in dystrophin exon 45–52 recapitulated the expected dystrophin deficiency (Lin et al., 2015). In addition, mitochondrial damage and increased levels of cell death were detected in the patients’ cells. Strikingly, Lin et al. (2015) were able to underpin this findings by identifying a mitochondria-mediated signaling network that linked damaged mitochondria with caspase-3 mediated apoptosis, thereby uncovering a potential new disease mechanism in DMD.

The iPSC-CM model has been also applied to examine the cardio-metabolic remodeling in type 2 diabetes. Modulation of the insulin signaling involves the tyrosine kinase receptor which activates insulin receptor substrate family proteins (IRS-1/2), phosphoinositide 3-kinase (PI3K) and AKT. In the heart, AKT activation promotes intracellular glucose import through translocation of the glucose transporter type 4 to the plasma membrane, thereby stimulating glycolysis and mitochondrial substrate oxidation (Abel, 2021). As a hallmark of type 2 diabetes mellitus, PI3K/AKT signaling is disrupted as a result of the insulin resistance, promoting a fatty acid oversupply (Wende and Abel, 2010). However, elevated intracellular fatty acid levels increase ROS production, thereby inducing cellular and mitochondrial damage, decreasing energy production and promoting lipid accumulation in a positive feedback loop (van de Weijer et al., 2011; Westermeier et al., 2016). In consequence, triglycerides, sphingolipids and ceramides accumulate in peroxisomes and liposomes, resulting in cellular oxidative stress and poor cardiac performance; a process called lipotoxicity (D'Souza et al., 2016). In fact, a large community-based sample study demonstrated a potential association of elevated circulating ceramide with lower left ventricle function, suggesting a tight correlation between cardiac performance and lipotoxicity (Nwabuo et al., 2019). A recent report demonstrated the impact of ceramide overload on the cellular physiology using iPSC-CMs (Bekhite et al., 2021). Herein, both diabetic-like culture conditions as well as SPTLC1 overexpression, which promotes *de novo* synthesis of ceramides, resulted in increased intracellular ceramide levels and lipid accumulation (Bekhite et al., 2021). Interestingly, these changes were associated with impaired PI3K/AKT signaling, transcriptional changes in metabolic enzymes, abnormal bioenergetic capacity, as well as an increase in ROS production and apoptosis. Further, cardiac lipotoxicity and oxidative stress induced mitochondrial fission (indicated by increased levels of DRP1 and MFF) as well as mitophagy (indicated by upregulation of PINK1 and LC3B) in iPSC-CMs, thereby providing novel insights in the pathological mechanisms underlying lipotoxic cardiomyopathy. Aligned to this findings, Drawnel and co-workers recapitulated the type 2 diabetes associated cardiomyopathy using a selective medium composition (Drawnel et al., 2014). Briefly, the group incubated iPSC-CMs with two different culture conditions: one group was cultured in standard maintenance medium, whereas the other one was exposed to glucose-free medium with addition of insulin and fatty acids (maturation media). Thereby, iPSC-CM cultures were forced to exclusively utilize fatty acids through a persistent insulin signaling modulation. After administration of diabetic factors that are typically found in blood plasma of diabetic patients (endothelin-1 and cortisol), a clear DCM phenotype in the patients’ iPSC-CMs was observed when incubated in a diabetogenic environment including sarcomeric disarrangements, lipid peroxidation, and decreased expression of Krebs cycle-associated enzymes (Drawnel et al., 2014).

Besides, primarily neurological diseases might have an impact on cardiac physiology, as it is the case in Huntington’s disease (Critchley et al., 2018). In order to understand the role of neuronal function and mitochondrial metabolism, neuronal stem cells with increasing polyglutamine (polyQ) repeat length in the huntingtin gene were engineered (Ghosh et al., 2020). Here, the expression of huntingtin exon 1 fragments with polyQ-expansions inducing a deleterious mitochondrial function, such as a lower mitochondrial respiration promoted by decreased Complex I and III activity. Interestingly, similar effects were described by Joshi and co-workers in iPSC-CMs (Joshi et al., 2019). In their study, iPSC-CMs with polyQ-expansions showed an aberrant mitochondrial network due hyperactivation of the mitochondrial fission complex, uncovering a potential phenotype correlation in Huntington’s disease.

**FIGURE 3:**
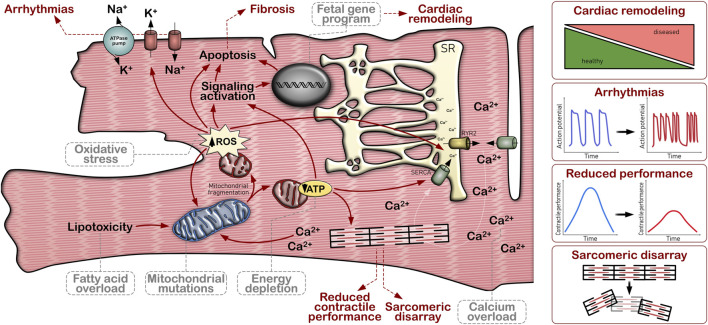
Impact of mitochondrial dysfunction on cardiomyocyte pathology. Mitochondrial dysfunction, either caused by gene mutations in mitochondrial genes (primary mitochondrial cardiomyopathy) or as a consequence of other cellular disturbances (such as calcium overload or lipotoxicity), promotes an energy depletion as well as increased ROS production. Poor bioenergetic capacity and oxidative stress provoke, among others, changes in cytosolic calcium handling, electrophysiological defects, reduced contractile performance, sarcomeric re-arrangements, re-expression of fetal genes, cardiac remodeling and apoptosis.

Summarizing, these examples highlight that maladaptive metabolic remodeling is not an exclusive pattern in mitochondrial cardiomyopathies Figure 3. Considering the high impact of mitochondrial function in multiple cellular processes, the bioenergetic modifications as a consequence of other forms of inherited cardiomyopathies should be taken into account in cardiovascular disease modeling.

## Drug Screening and Preclinical Gene Therapy Approaches for Mitochondrial Cardiomyopathies

Although several therapeutic strategies using iPSC-CMs has been raised over the past decade, the limited number of disease models for mitochondrial disorders hampers drug screening approaches and represents one of the major hurdles in the development of novel treatments for mitochondrial cardiomyopathies. Nevertheless, recent efforts in iPSC-based drug screening had been reported. Further, significant breakthroughs in genome editing technologies enabling genetic correction of underlying gene mutations show great promise for the therapy of mitochondrial disorders.

A major upgrade that the iPSC technology offers is to screen selective therapeutics under more physiological conditions in patient-derived and disease-relevant cells, tissues or even organ-like cultures. For instance, to assess the efficacy of treatment for Friedreich’s ataxia, a pool of different antioxidants (an iron chelating agent deferiprone and an ubiquinone-derived analog idebenone) were tested in frataxin-deficient iPSC-CMs mimicking the cardiac stress stimulation (Lee et al., 2016). In their study, ROS production was evaluated after antioxidants administration in presence and absence of iron. Interestingly, deferiprone significantly decreased the intracellular ROS levels promoted by iron overload, and restored some aspects of deleterious Ca^2+^ handling in the patients’ iPSC-CMs, suggesting a tight correlation between iron overload-induced mitochondrial oxidative stress and cardiomyocyte contractility (Lee et al., 2016).

A drug screening approach in patients’ iPSC-CMs had been also performed for DCM as a result of TIM14 gene loss-of-function; the respective disease model had been introduced in the previous chapter (Rohani et al., 2020). Although, a severe disruption of the mitochondrial morphology was observed in TIM14-deficient cells, most of the deleterious effects could be restored by elamipretide treatment that resulted in a profound reduction of the proteolytic cleavage of the short OPA1 isoform, in lower levels of fragmentation of the mitochondrial network, as well as in an improved mitochondrial ultrastructure (Rohani et al., 2020). This inner mitochondrial membrane-targeting peptide is currently used in clinical trials for patients with primary mitochondrial myopathies (Karaa et al., 2020; Reid Thompson et al., 2021).

Further, iPSC models for type 2 diabetes were also utilized for compound screening. An impressive library of 480 compounds was tested in patient-specific iPSC-CMs and 10 out of these small molecules were able to restore the pathological phenotype. Here, thapsigaragin and fluspirilene presented the most-effective impact in cells under diabetic stress (Drawnel et al., 2014). Bekhite and co-workers explored the potential role of myriocin (a potent sphingosine biosynthesis inhibitor) in their SPTLC1 overexpression iPSC-CM model. Interestingly, most of the deleterious effects observed by ceramide accumulation—predominantly, fission morphology and mitochondrial/lysosome co-localization—was restored by myriocin. Moreover, mitochondrial respiration was significantly upregulated after SP1 inhibitor treatment (Bekhite et al., 2021). These examples demonstrate that iPSCs can be widely used for high-content phenotypic screenings for the estimation of drug effectiveness, toxicology and drug metabolism, thereby taken in consideration the patients’ genetic background as well as external pathophysiological factors.

On the opposite side, gene therapy as a consequence of the rapidly developing programmable genome editing tools may offer a promising resource to bypass the hurdles in the treatment of mitochondrial disorders. Particularly, huge progress in CRISPR/Cas9 genome editing and base editing for genetic correction of nuclear DNA gene mutations—such as for DMD (Amoasii et al., 2018) or for the Hutchinson-Gilford syndrome (Koblan et al., 2021)—can be transferred practically one-to-one to nuclear DNA-associated mitochondrial disorders. To name a few: Friedreich’s ataxia and coenzyme Q10 deficiency were successfully corrected by genome editing *in vitro*. For the former, Li and co-workers utilized two zinc finger nucleases specifically targeting the intronic region of GAA repeats in the frataxin gene (Li et al., 2019). As a result, the repeat expansion sequence was reduced by ∼1.2 kbps and total protein levels of full-length functional frataxin were doubled. In consequence, lipid droplets accumulation and expression of cardiac stress markers typically observed in untreated patients’ iPSC-CMs were restored after zinc finger nuclease-based treatment (Li et al., 2019).

For coenzyme Q10 deficiency, Nakamoto and co-workers applied a site-specific CRISPR/Cas9-mediated gene correction and evaluated its subsequent molecular and functional characteristics (Nakamoto et al., 2018). In detail, coenzyme Q10 represents one of the major antioxidant factors for the MRC, and its synthesis is commanded by coenzyme Q2 (polyprenyltransferase; COQ2) which catalyzes the second step of coenzyme Q10 biosynthesis (Forsgren et al., 2004; Quinzii et al., 2006). In the present study, the group generated iPSCs from a patient that harbors compound heterozygous mutations in the COQ2 gene (p.R387*/V393A) and observed an aberrant mitochondrial respiration, an elevated ROS production and increased apoptotic levels in iPSC-derived neuronal cells. Interestingly, after CRISPR/Cas9-based correction of both gene mutations on both alleles, the pathological parameters were fully restored, highlighting the promise of the genome editing technologies for future clinical translation.

In strong contrast, CRISPR/Cas9-based genome editing of mtDNA represents a significantly bigger challenge, mainly because of the absence of efficient endogenous RNA import mechanisms that would be required to introduce the CRISPR guide RNA into the mitochondrial matrix (Gammage et al., 2018a). This disadvantage motivated to develop alternative protein-only genome editing tools fused to a mitochondrial targeting signal, which enable to selectively modify mtDNA. Although these mitochondrial-specific transcription activator-like effector nucleases (mitoTALEN), zinc finger nucleases (mtZFN) and meganucleases (mitoARCUS) have not yet been tested in the human cardiac setting, these systems demonstrated certain success in selective degradation of mutant mtDNA by nuclease-mediated cleavage of mtDNA in both patients’ iPSCs (Yahata et al., 2017; Yang et al., 2018) as well as *in vivo* (Gammage et al., 2018b; Zekonyte et al., 2021), thereby resulting in heteroplasmic shifts by decreasing the pathological heteroplasmy levels. Importantly, classical DNA repair mechanisms are limited in mitochondria (Fu et al., 2020), which promotes major effects in edited cells, such as: 1) mitochondrial repair pathways of introduced double stand breaks in the mtDNA typically result in deletions that can manifest in a pathological phenotype, 2) nuclease-induced mtDNA linearization is rapidly recognized leading to rapid degradation of mtDNA and depletion of mitochondria (Peeva et al., 2018), and 3) ineffective mtDNA repair may trigger a toxic accumulation of mitochondrial content in host cells, therefore, might enhance intrinsic immune surveillance mechanisms (Tigano et al., 2021). This major hurdle can be overcome by precise base editing without induction of double strand breaks. Very recently, Mok and co-workers engineered a mitochondrial RNA-free base editor DdCBE by fusion of transcription activator-like effector proteins with split versions of a double-stranded deaminase DddA_tox_ to induce selective C to T conversion in mtDNA with high target specificity and product purity (Mok et al., 2020). Strikingly, the authors confirmed that base editing (tested in HEK293T cells) was almost exclusively observed on the target sites at various mtDNA-encoded gene loci with an efficiency of up to 27%, becoming a promising approach in the future to precisely correct mtDNA variants and to decrease the mtDNA heteroplasmy (Mok et al., 2020). However, as it is true for the majority of gene therapy approaches, efficient delivery of the genome editing components into target organs, target cells and mitochondria remains currently one of the biggest challenges for clinical translation.

Finally, although not applicable as therapy for patients with mtDNA disorders, it should be mentioned that mitochondrial DNA replacement technologies aiming to transfer nuclear DNA from a zygote containing disease-causing mtDNA variants to a corresponding zygote with healthy mitochondria, offer a huge therapeutic benefit for assisted reproductive medicine to avoid pathogenic mtDNA transmission between generations (Greenfield et al., 2017).

In summary, the high versatility of iPSC-CMs have proven a powerful pre-clinical platform in the screening of drugs and genome editing tools in a patient context and might support the translation of these approaches into clinical practice by bridging the gap between preclinical and clinical research.

## Challenges and Limitations of iPSC-CMs for Mitochondrial Cardiomyopathy Research

Mature cardiomyocytes reflect a very particular set of properties that involve myofibril re-arrangements, characteristic electrophysiological patterns, increased contractility, decreased cell cycle progression and a metabolic shift (Guo and Pu, 2020). The metabolic state is central in cardiac development and multiple studies demonstrated that a distinct substrate oxidation is present during the process of cardiac maturation (Lopaschuk and Jaswal, 2010; Dorn et al., 2015). The fetal heart exhibits a high glycolytic metabolism, accompanied by expression of different glucose transports and distinct transcriptional factors that maintain the glycolytic program in immature cardiomyocytes (Taegtmeyer et al., 2010). In a remarkable shift during postnatal development, around 80% of ATP production in the adult heart is generated from fatty acids oxidation and OXPHOS activity (Wisneski et al., 1987; Koff and Schimmel, 1993). This massive change in metabolic supply is mainly explained as a consequence of substrates availability, metabolic pathway activity and differential transcriptional factor induction (Lopaschuk and Kelly, 2008). Indeed, the metabolic transition from immature cardiomyocytes to mature cardiomyocytes is driven by the activation of transcriptional regulators including PGC-1α/β and NRF1/2, resulting in upregulation of metabolic genes that are involved in fatty acid transport and oxidation, OXPHOS as well as mitochondrial biogenesis (Lai et al., 2008; Leone and Kelly, 2011; Vega and Kelly, 2017). Simultaneously, a downregulation in glycolytic genes, among others, mediated by HIF-1α inactivation, enhances the switch towards mitochondrial metabolism (Menendez-Montes et al., 2016). A considerable rearrangement in mitochondrial morphology orchestrated by fusion/fission proteins is necessary for an optimal mitochondrial bioenergetic adaptation. As an example, mitofusion 1/2 and OPA1 that regulate the fusion of external and internal mitochondrial membranes, are increased in mature cardiomyocytes, promoting an effective ATP generation (Martin et al., 2014). Furthermore, an increased coordination among sarcoplasmic reticulum, sarcomeres and mitochondria has been described during cardiac maturation (Seppet et al., 2001; Guo et al., 2021). In depth, adult cardiomyocytes show a highly structured sarcomere pattern associated with an abundant mitochondrial network, which, in turn, presents several contact sites with the sarcoplasmic reticulum. This close interaction leads to efficient ATP transport from mitochondria to ATPases that participate in excitation-contraction coupling (Seppet et al., 2001).

Although recent studies have reported iPSC-CMs as a suitable experimental model for mitochondrial disease (see section three), one of the major limitations of these cells is their fetal-like properties compared to adult cardiac tissue, specifically their poor mitochondrial bioenergetic capacity (Guo and Pu, 2020). This limitation might have remarkable relevance in disease modeling particularly of mitochondrial disorders, since less-mature (glycolytic) iPSC-CMs might not reproduce the expected pathological disease phenotype. Regarding this issue, numerous methods have been developed in order to facilitate iPSC-CM maturation to generate an “adult-like” phenotype. Most of these attempts can be grouped in delivery of energy sources (e.g. fatty acids versus glucose), biochemical cues, transcriptional factor modulation, physical/electrical stimulation and 3D tissue engineering. Herein, we aim to summarize some of these strategies with particular emphasis on cardiac metabolism and mitochondrial function. One of the first studies that proposed fatty acid-based conditioned medium to induce cardiomyocyte maturation was provided by Correia and co-workers using a mixture of fatty acids (oleic acid, palmitate, and galactose) (Correia et al., 2017). After approximately 2 weeks of incubation, iPSC-CMs showed elevated mitochondrial respiration attended by elongated cell morphology, higher organized sarcomeric structures and an increased action potential upstroke velocity, comparable to adult cardiomyocytes (Correia et al., 2017). A subsequent study demonstrated that iPSC-CMs incubated in fatty acid-based medium showed an increased ATP generation as a consequence of elevated mitochondrial OXPHOS activity, resulting in increased force generation and contractility (Yang et al., 2019). Since then, many other studies have used this approach to evaluate different aspects of cardiomyocyte differentiation. For instance, Knight and co-workers used a similar conditioned medium combined with substrate patterning and could confirm that iPSC-CMs displayed an elongated morphology, a higher sarcomeric alignment and an elevated contraction force, equivalent with donor heart tissue (Knight et al., 2021). Besides, a differential and more robust pathological hypertrophic phenotype could be observed using the aforementioned setting. In line, fatty acid-based media also allowed a phenotypic resemblance of different inherent cardiomyopathies with more-mature iPSC-CMs, such as arrhythmogenic episodes described in Long QT syndrome, dilated cardiomyopathy or Danon disease (Feyen et al., 2020; Knight et al., 2021). Collectively, considering the high impact of substrate oxidation in mitochondrial function, the selective composition of culture media should be taken into consideration when studying the wide spectrum of phenotypic changes induced by mitochondrial disorders.

Considering the high prevalence that glycolytic metabolism plays at embryonic stage, an alternative cardiomyocyte maturation strategy involved the downregulation of glycolysis-associated components by inactivation of the hypoxia-inducible factor 1-alpha (HIF-1α) and the lactate dehydrogenase A (LDHA) during cardiac differentiation of iPSCs (Hu et al., 2018). Whereas HIF-1α controls glycolytic gene expression during hypoxic conditions and suppresses mitochondrial metabolism, LDHA mediates the conversion of pyruvate into lactate, thereby decreasing pyruvate oxidation in the mitochondria (Kim et al., 2006; Semenza, 2007). Hu and coworkers used small molecule inhibitors and small interfering RNAs (siRNAs) against HIF-1α and LDHA and observed reduced glycolytic activity and enhanced mitochondrial metabolism that was accompanied by increased mtDNA levels and mitochondrial content (Hu et al., 2018). Strikingly, HIF-1α/LDHA inhibition in iPSC-CMs did not only provided metabolic maturation, it also improved structural and functional maturation (Hu et al., 2018). Although this setting has not yet been tested in pathological conditions using iPSC-CMs, the metabolic shift from aerobic glycolysis to mitochondrial OXPHOS may be useful to understand the cardiac remodeling associated with mitochondrial insufficiencies.

Simultaneously, circulating factors and drugs have been tested as potential inductors of iPSC-CM maturation. Triiodothyronine (T3) is one of the most relevant metabolic hormones controlling the mitochondrial biogenesis and metabolism through its regulation via the thyroid hormone receptor-mediated pathway (Goldenthal et al., 2005). T3 has a preponderant effect on cardiomyocyte development, supported by the fact that cardiac defects have been described in congenital hypothyroidism patients (Wędrychowicz et al., 2019). Yang et al. (2014) showed that iPSC-CM cultures treated with T3 for over 1 week presented an augmentation of cellular size and sarcomere length and increased force generation. Interestingly, T3 treatment decreased cell cycle activity, an attribute also described in adult cardiomyocytes. Additionally, all these changes were aligned with a significant enhancement of mitochondrial function Yang et al. (2014). Thereafter, Parikh et al. (2017) demonstrated that combined treatment with T3 and glucocorticoids during cardiac differentiation resulted in an increase of cellular size and improved contractility. Surprisingly, iPSC-CMs also developed an extensive T-tubule network when using a Matrigel mattress. Although, the T-tubules were less structured compared to adult human myocardium, the distribution of junctophilin-2, which is mainly associated with T-tubule maturation, was significantly increased after T3 and dexamethasone treatment (Parikh et al., 2017). At the same time, iPSC-CMs showed a uniform Ca^2+^ release, a characteristic of maturated “adult-like” cardiomyocytes. In line, an iPSC-CM disease model based on a MYBPC3 gene mutation causing HCM revealed a robust disease phenotype only in maturation medium containing T3, IGF-1 and dexamethasone (Birket et al., 2015). In analogy to hormones, drug-based targeting of the AMP-activated protein kinase (AMPK), one of the master metabolic regulators of the cell (Garcia and Shaw, 2017), have also been tested to enhance mitochondrial respiration and to induce maturation. AMPK subunit gamma acts a sensor for adenosine monophosphate (AMP) and participates in the catalytic activation of the enzyme. Thus, when AMP/ATP ratio is high, AMPK is activated, thereby increasing the energy production through modulation of various signaling cascades (Garcia and Shaw, 2017). Two recent independent reports probed that AMPK modulation induced maturation of iPSC-CMs (Sarikhani et al., 2020; Ye et al., 2021). Long-term incubation of cultures with AMPK activator AICAR increased the expression of mitochondrial biogenesis-related markers such as PGC-1α and ERRα, increased fatty acid β-oxidation, and displayed an elevated mitochondrial density as well as an extended mitochondrial network. Besides, these changes were accompanied by increased expression of sarcomeric proteins including cardiac troponin T and cardiac troponin I as a consequence of long-term AICAR treatment.

Finally, 3D-engineered cardiac tissue, composed of iPSC-CMs and non-myocytes embedded in a hydrogel, has been considered the gold standard with respect to structural and functional maturation by more closely reflecting the native physiological conditions of the human heart muscle (Tiburcy et al., 2017). Indeed, the improved contractile properties of these cardiac tissues also contributed to a metabolic maturation of iPSC-CMs with higher abundance of mitochondria, less anaerobic glycolysis and an improved oxidative metabolism (Ulmer et al., 2018). In an extensive work performed by Ronaldson-Bouchard et al. (2018) addressing the maturity aspects of iPSC-CMs, they could demonstrate a robust sarcomeric re-arrangement, T-tubule maturation, increased contractility, and enhanced mitochondrial energy production upon early-stage biophysical pacing of cardiac tissues, highly recapitulating an “adult-like” cardiac phenotype.

Collectively, the physiologically immature state of iPSC-CMs might be a critical limitation in cardiac disease modeling by masking disease-relevant phenotypes/symptoms such as the disease-associated bioenergetic mitochondrial function. An improved maturation of iPSC-CM cultures accompanied with a shift from aerobic glycolysis to OXPHOS metabolism (e.g. by using one of the aforementioned approaches) might significantly improve evaluation of the cardiac pathology *in vitro* and should be taken into account in iPSC-based disease modeling of mitochondrial disorders.

## Conclusion

Mitochondrial diseases can promote multi-organ insufficiency during early or late state. Around one third of these patients present a type of cardiac involvement by showing the poorest prognosis in the childhood. However, the lack of suitable disease models and the challenges associated with the different levels of heteroplasmy have been one of the major hurdles in the discovery of therapeutic targets. Our knowledge of iPSC biology has advanced to the point where we now can generate the majority of disease-relevant cell types of the human body in a culture dish, promoting iPSC technology as a suitable tool to overtake these challenges. The great variability of cardiac symptoms associated to mitochondrial disorders can by now be recapitulated and modelled more precisely, helping to evaluate tissue-specific threshold of mtDNA mutation load for individual genes or gene variants as well as to elucidate the tentative mechanisms behind the metabolic perturbations promoted as a consequence of mitochondrial disorders. Considering the high impact of maturity of iPSC-CMs in mitochondrial metabolism, it becomes crucial to not only study the pathology in traditional 2D culture systems, but to also incorporate improved maturation methods in order to get the most representative cardiac phenotype-genotype correlation. These efforts might be central to develop novel and personalized therapeutic approaches.
